# Oncogenic role of the SLC7A13-SLC3A1 cystine transporter in human luminal breast cancer and its cryo-EM structure

**DOI:** 10.1093/procel/pwaf076

**Published:** 2025-09-06

**Authors:** Jing Dong, Tianhao Shi, Bingbiao Lin, Xuetong Liu, Waner Wei, Zichi Geng, Mingcheng Liu, Renhong Yan, Jin-Tang Dong

**Affiliations:** Department of Human Cell Biology and Genetics, SUSTech Homeostatic Medicine Institute, School of Medicine, Southern University of Science and Technology, Shenzhen 518055, China; Department of Biochemistry, SUSTech Homeostatic Medicine Institute, School of Medicine, Key University Laboratory of Metabolism and Health of Guangdong, Institute for Biological Electron Microscopy, Southern University of Science and Technology, Shenzhen 518055, China; Department of Human Cell Biology and Genetics, SUSTech Homeostatic Medicine Institute, School of Medicine, Southern University of Science and Technology, Shenzhen 518055, China; Department of Radiotherapy, Cancer Hospital of Shantou University Medical College, Shantou 518055, China; Department of Human Cell Biology and Genetics, SUSTech Homeostatic Medicine Institute, School of Medicine, Southern University of Science and Technology, Shenzhen 518055, China; Department of Human Cell Biology and Genetics, SUSTech Homeostatic Medicine Institute, School of Medicine, Southern University of Science and Technology, Shenzhen 518055, China; Department of Human Cell Biology and Genetics, SUSTech Homeostatic Medicine Institute, School of Medicine, Southern University of Science and Technology, Shenzhen 518055, China; Department of Human Cell Biology and Genetics, SUSTech Homeostatic Medicine Institute, School of Medicine, Southern University of Science and Technology, Shenzhen 518055, China; Department of Biochemistry, SUSTech Homeostatic Medicine Institute, School of Medicine, Key University Laboratory of Metabolism and Health of Guangdong, Institute for Biological Electron Microscopy, Southern University of Science and Technology, Shenzhen 518055, China; Department of Human Cell Biology and Genetics, SUSTech Homeostatic Medicine Institute, School of Medicine, Southern University of Science and Technology, Shenzhen 518055, China

**Keywords:** cryo-EM, breast cancer, SLC7A13, cystine transporter, ferroptosis

## Abstract

Breast cancer is a prevalent malignancy worldwide. The majority of breast cancers belong to the estrogen receptor (ER)-positive luminal subtype that can be effectively treated with antiestrogen therapies. However, a significant portion of such malignancies become hormone-refractory and incurable. Cancer cells often uptake more cystines to increase glutathione (GSH) biosynthesis and reduce reactive oxygen species (ROS), thereby preventing ROS-induced ferroptosis and leading to therapeutic resistance. However, few molecules of these processes are targetable for cancer therapy. However, few therapeutic targets have been established that target these processes. Here, we report that the gene for SLC7A13, a member of the SLC7A13-SLC3A1 cystine transporter, was amplified and overexpressed in 19.7% and 49.7% of breast cancers, respectively. *SLC7A13* amplification and overexpression were associated with worse overall survival and disease-free survival in patients with luminal breast cancer. Functionally, *SLC7A13* overexpression promoted, while its silencing attenuated, cell survival or proliferation. Molecularly, *SLC7A13* silencing reduced cystine uptake and GSH biosynthesis, leading to increased lipid ROS levels. The cryo-EM structure of the human SLC7A13-SLC3A1 complex was determined at 2.64 Å, revealing a dimer-of-heterodimers architecture similar to that of other SLC3A1-linked transporters. A specific substrate-binding pocket was identified, containing distinct residues, which suggests a regulatory role in the cystine transporter. These findings suggest that the SLC7A13-SLC3A1 cystine transporter is a therapeutic target for treating luminal breast cancer. They also provide the structural insights for therapeutic development targeting the cystine transporter.

## Introduction

Ferroptosis is a form of cell death induced by the accumulation of lipid peroxides in cell membranes (Dixon et al., 2012; Jiang et al., 2021). Cancer cells often develop various mechanisms to overcome ferroptosis; therefore, inducing ferroptosis is considered a therapeutic approach in cancer treatment (Dixon et al., 2012; Tan et al., 2001). In mammalian cells, glutathione (GSH) is the most prevalent antioxidant, and glutathione peroxidase 4 (GPX4) is the primary enzyme that catalyzes the reduction of membrane phospholipid hydroperoxides, as GPX4 transforms GSH into oxidized glutathione, thereby detoxifying lipid peroxides into lipid alcohols (Maiorino et al., 2018). GSH is a tripeptide composed of cysteine, glutamate, and glycine. Therefore, the availability of cysteine is a critical factor in GSH biosynthesis (Meister, 1995), and the cyst(e)ine-GSH-GPX4 axis is considered a primary mechanism that regulates ferroptosis (Yang et al., 2014), offering a promising opportunity for therapeutic development.

Cellular cysteine level is maintained by cystine uptake via its transporters or *de novo* synthesis from serine and homocysteine. Due to excess oxidative stress, tumor cells demand more cysteine for GSH biosynthesis to maintain lower levels of reactive oxygen species (ROS) and enhanced cell survival (Trachootham et al., 2009). However, cysteine synthesis is either inactive or downregulated in tumors, making cystine uptake more crucial for maintaining the tumor cells’ cysteine pool (McBean, 2002; Yoon et al., 2023). Indeed, inhibition of cystine transporter activity leads to the depletion of intracellular GSH, the subsequent inactivation of GPX4, and ferroptosis (Banjac et al., 2008; Miura et al., 1992). Even in normal tissues, depletion of the extracellular L-cysteine and cystine pool leads to cell cycle arrest and cell death, as demonstrated in mice and non-human primates (Cramer et al., 2017).

Cystine transporters are heterodimeric amino acid transporters (HATs) consisting of a light chain subunit from the SLC7 family and a heavy chain subunit from the SLC3 family (Palacín and Kanai, 2004; Palacín et al., 1998). The light chains act as the transport subunits, while the heavy chains ensure the membrane localization and functionality of HATs (Nagamori et al., 2016). Two heavy chains have been identified, including SLC3A1 (also known as rBAT) and SLC3A2 (also known as 4F2hc or CD98hc) (Verrey et al., 2000). The SLC7A11-SLC3A2 transporter (system xc^−^, formed by SLC3A2/4F2hc and SLC7A11/xCT) is considered a potential therapeutic target in cancer treatment because inhibiting this transporter’s activity reduces cystine uptake and causes ROS accumulation and subsequent ferroptosis (Badgley et al., 2020; Floros et al., 2021; Ji et al., 2018; Tang et al., 2023).

Another heavy chain subunit of cystine transporters, SLC3A1 (also known as rBAT), dimerizes with either SLC7A9 (also known as b^0,+^AT) or SLC7A13 (also known as AGT1) to transport cystine (Boutros et al., 2005; Matsuo et al., 2002; Nagamori et al., 2016; Yuen et al., 2006). However, it is unknown whether these two cystine transporters impact tumorigenesis. Recent high-resolution cryogenic electron microscopy (cryo-EM) structures of SLC7A9-SLC3A1 and SLC7A11-SLC3A2 have revealed their substrate recognition and transport mechanism (Parker et al., 2021; Yan et al., 2020, 2022b), but that for the SLC7A13-SLC3A1 transporter is unavailable.

In this study, we evaluated the genetic alterations of *SLC7A13* in human breast cancer and tested the effect of *SLC7A13* silencing on breast cancer cells. We found that *SLC7A13* was frequently amplified and upregulated in breast cancers, and elevated *SLC7A13* expression enhanced cellular behaviors indicative of tumor malignancy. *SLC7A13*’s silencing also increased lipid ROS levels by reducing cystine uptake in breast cancer cells. We also determined the cryo-EM structure of the SLC7A13-SLC3A1 transporter complex and identified several key residues within the substrate pocket.

## Results

### *SLC7A13* amplification and overexpression in aggressive luminal breast cancer

**Figure 1.**
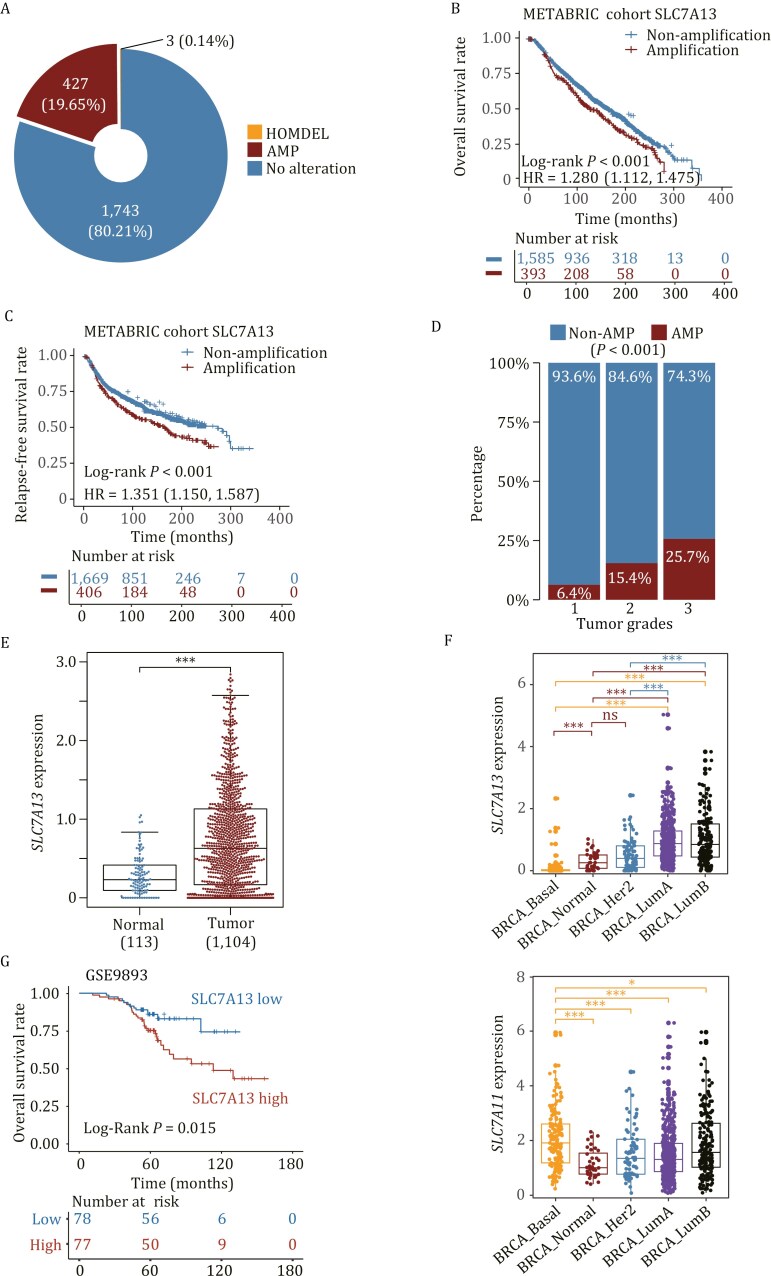
**Amplification and overexpression of *SLC7A13*/*AGT1* and their association with poor survival in breast cancer.** (A) Gene amplification is the major form of alteration in *SLC7A13* in breast cancer samples, as analyzed in the METABRIC cohort. AMP is gene amplification; HOMDEL is homozygous deletion. (B and C) Amplification of *SLC7A13* significantly reduced the overall survival (B) and the relapse-free survival (C) of breast cancer patients, as determined using the Kaplan–Meier analysis in the METABRIC cohort. (D) *SLC7A13* amplification correlates with higher tumor stages in the METABRIC breast cancer cohort. (E) *SLC7A13* mRNA expression was significantly higher in breast cancer than in normal breast tissues, as analyzed using the TCGA breast cancer dataset. (F) Expression of *SLC7A13* (upper) and *SLC7A11* (lower) in different breast cancer subtypes, as analyzed using the TCGA breast cancer dataset. (G) Higher *SLC7A13* levels were significantly correlated with the overall survival of breast cancer patients, as analyzed using the GSE9893 dataset.

Among 2,173 profiled breast cancer samples in the METABRIC cohort, *SLC7A13* was amplified in 427 (19.7%) cases (Fig. 1A). Kaplan–Meier analysis revealed that patients with *SLC7A13* amplification had significantly shorter overall survival (Log-rank *P* < 0.001) (Fig. 1B, upper panel) and relapse-free survival (Log-rank *P* < 0.001) (Fig. 1C, upper panel). Univariate Cox regression analyses also demonstrated a significant correlation of *SLC7A13* amplification with worse overall survival (HR: 1.280, 95% CI: 1.112‒1.475) and relapse-free survival (HR: 1.351, 95% CI: 1.150‒1.587) (Fig. 1B and 1C, lower panel). Additionally, *SLC7A13* amplification correlated with higher histological grades in breast cancer, as determined using the Fisher exact test (*P* < 0.001, Fig. 1D). Analysis of the TCGA database demonstrated that breast cancers expressed significantly higher mRNA levels of *SLC7A13* than normal breast tissues (*P* < 0.001, Fig. 1E). In fact, 549 out of 1,104 breast cancers (49.73%) expressed *SLC7A13* at levels higher than twice of normal tissues’ average *SLC7A13* level (Fig. S1E).

When the breast cancers were divided into four major subgroups, including basal, HER2, luminal A, and luminal B, *SLC7A13* mRNA level was significantly higher in luminal A and luminal B tumors compared to normal tissues (Fig. 1F, upper panel). Meanwhile, the *SLC7A13* levels in basal tumors were significantly lower than those in luminal tumors (Fig. 1F, upper panel). Unexpectedly, the *SLC7A13* mRNA level in basal tumors was even lower than in normal tissues (Fig. 1E, upper panel). In the GSE9893 dataset, a higher median mRNA level of *SLC7A13* was significantly associated with worse overall survival (Log-rank *P* = 0.015) (Fig. 1G). These findings suggest that elevated *SLC7A13* expression plays a role in the progression of luminal breast cancer but is not significantly involved in basal tumors.

We conducted a similar analysis for SLC7A11, another member of the cystine transporter subunits that has been established as a therapeutic target in the basal subtype of breast cancers (Timmerman et al., 2013). *SLC7A11*’s expression pattern differed from *SLC7A13*’s, as *SLC7A11* mRNA level was higher in basal tumors than in luminal tumors and normal breast tissues (Fig. 1F, lower panel).

The estrogen/ER signaling is abnormally active in luminal breast cancers, and antiestrogen therapy is thus commonly used to treat this subtype of breast cancer. Approximately 50% of breast cancers significantly overexpressed SLC7A13, whereas about 20% of them exhibited *SLC7A13* amplification (Figs. 1A and S1E), suggesting that other mechanisms also contribute to *SLC7A13* overexpression in luminal breast cancers. We tested whether *SLC7A13* transcription responds to the estrogen/ER signaling activity. In the ER-positive T-47D breast cancer cells, E_2_ (estradiol) increased *SLC7A13* expression in a dose-dependent manner but did not affect *SLC7A11* expression in such a manner (Fig. S2A). In addition, the estrogen signaling pathway was among the significantly enriched pathways in breast cancers with higher *SLC7A13* mRNA levels; however, this was not the case for *SLC7A11* (Fig. S2B). Moreover, higher estrogen activities significantly correlated with higher *SLC7A13* mRNA levels in breast cancers (*R* = 0.486, *P* < 2.2 × 10^−16^) but did not correlate with that of *SLC7A11* (*R* = 0.025, *P* = 0.372) (Fig. S2C). Therefore, increased ER activity in luminal breast cancers should also contribute to elevated SLC7A13 expression in these cancers.

### Elevated *SLC7A13* expression enhances malignant behaviors of breast cancer cells

**Figure 2.**
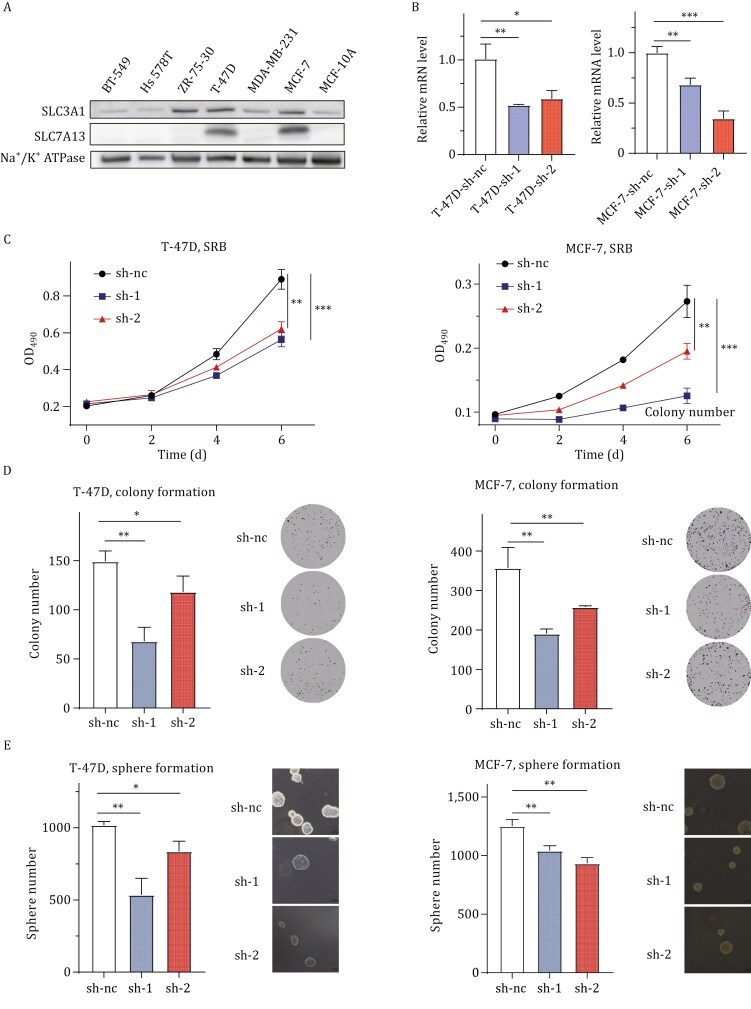
**SLC7A13 enhances the proliferation of breast cancer cells.** (A) Expression of SLC7A13 and its partner SLC3A1 in 7 breast cancer cell lines, as detected by Western blot. Na^+^/K^+^ ATPase was used as the loading control. (B) Validation of *SLC7A13* knockdown by lentiviral shRNAs in T-47D and MCF-7 breast cancer cell lines after infection, as detected by real-time qPCR. (C‒E) Knockdown of *SLC7A13* in T-47D and MCF-7 cell lines inhibited cell proliferation (C), colony formation (D), and sphere formation in soft agar (E). Panels D and E include representative images and data quantification. Each data point had three replicates (C‒E). Data are presented as mean ± SD. *, *P* < 0.05; **, *P* < 0.01; ***, *P* < 0.001.

To determine whether SLC7A13 plays a promoting role in breast cancer, we surveyed 7 breast cancer cell lines. We found that *SLC7A13* was expressed at higher levels in MCF-7 and T-47D cell lines (Fig. 2A). The gene encoding SLC7A13’s partner, *SLC3A1*, was expressed at relatively constant levels in 5 of the 7 cell lines (Fig. 2A). *SLC7A13* was then knocked down in T-47D and MCF-7 cell lines and evaluated for its impact on cell behaviors indicative of malignancy. The transfection efficiency in both MCF-7 and T-47D cells was relatively poor, so lentiviral shRNA was used to knock down *SLC7A13* (Fig. 2B). As demonstrated in Fig. 2, *SLC7A13* silencing significantly reduced cell numbers in both cell lines, as detected using the SRB assay (Fig. 2C). *SLC7A13* silencing also significantly attenuated colony formation on a plastic surface (Fig. 2D) and anchorage-independent growth, as indicated by sphere formation in soft agar (Fig. 2E). Therefore, increased expression of *SLC7A13* enhances malignant behaviors of breast cancer cells.

### SLC7A13 overexpression increases cystine uptake to reduce ROS and ferroptosis

**Figure 3.**
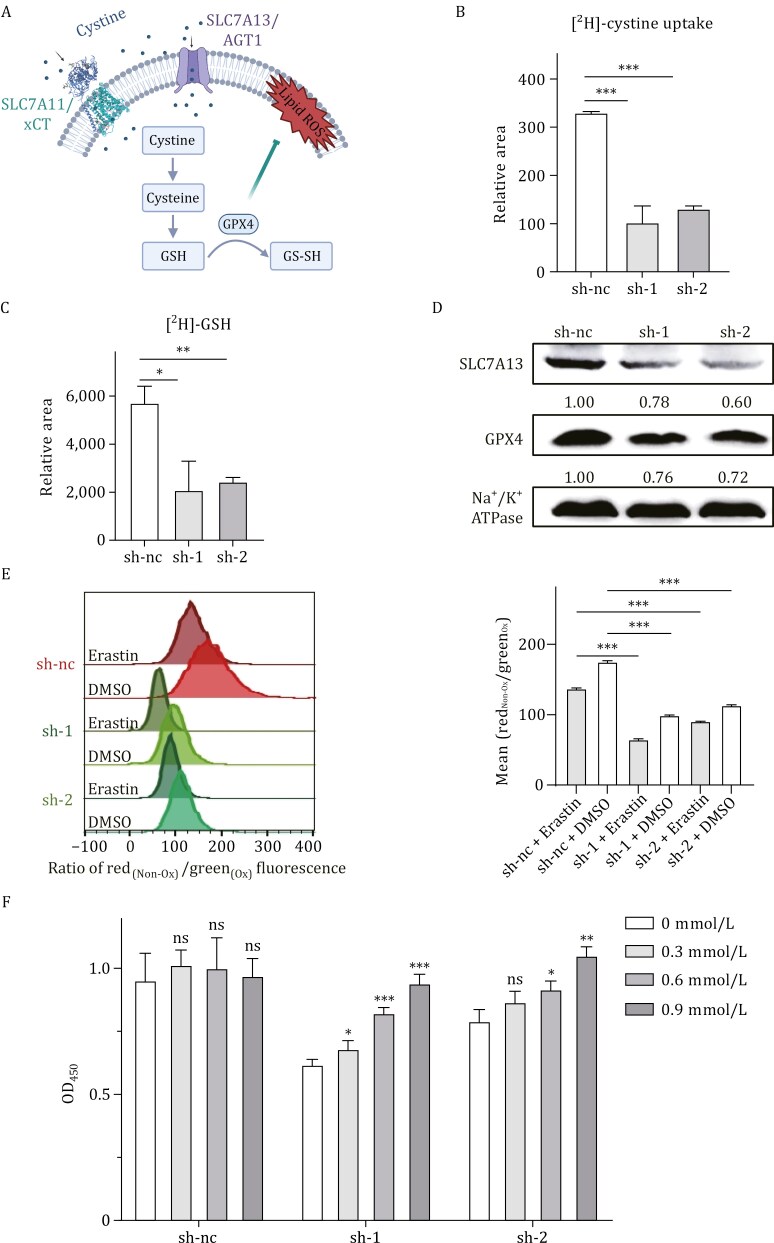
**SLC7A13 maintains cysteine uptake to reduce ROS levels in breast cancer cells.** (A) A schematic was created using BioRender.com to illustrate how the SLC3A1-SLC7A13 transporter transports cystine into cells, thereby reducing lipid peroxidation on the cell membrane. (B and C) Knockdown of *SLC7A13* augmented cystine uptake (B) and GSH synthesis (C), as determined using the isotope ratio mass spectrometry assay in T-47D breast cancer cells. (D) Knockdown of SLC7A13 reduced GPX4 expression in T-47D breast cancer cells, as detected by Western blot. (E) *SLC7A13* silencing elevated cellular ROS levels in T-47D cells, as detected by staining lipid peroxidation using C11-BODIPY. Erastin is a ROS inducer and was used as a positive control. (F) *SLC7A13* silencing induced cell death, whereas cystine supplementation reversed this effect in T-47D cells, as determined by the CCK8 assay. Data are presented as mean ± SD. *n* = 3. *, *P* < 0.05; **, *P* < 0.01; ***, *P* < 0.001.

Cysteine or cysteine deprivation leads to the accumulation of ROS. Increased ROS levels induce ferroptosis and thus suppress tumor growth (Fig. 3A). Overexpression of another cystine transporter subunit, SLC7A11, has been demonstrated to reduce ROS levels and inhibit ROS-induced ferroptosis (Armenta et al., 2022; Cramer et al., 2017; Jiang et al., 2015). We therefore tested whether elevated *SLC7A13* expression has similar effects in breast cancer cells. In T-47D breast cancer cells, which expressed a higher level of SLC7A13 (Fig. 2A), mass spectrometric analysis with isotope tracking demonstrated that *SLC7A13* silencing significantly reduced the uptake of cysteine (Fig. 3B). Consistently, *SLC7A13* silencing also decreased the GSH level in the same cells (Fig. 3C). GPX4 is downregulated upon GSH depletion during ferroptosis induced by reducing cystine uptake (Yang et al., 2014). *SLC7A13* silencing also downregulated GPX4 protein level in T-47D cells (Fig. 3D). Compared to the positive control erastin, a potent SLC7A11 blocker that significantly increased ROS in the shRNA control group, *SLC7A13* silencing with each shRNA also increased lipid ROS levels when compared to the shRNA control (Fig. 3E). In addition, combined erastin treatment and SLC7A13 knockdown had an additive effect on ROS induction for each SLC7A13 shRNA (Fig. 3E).

To further investigate whether cystine uptake influences cell death upon *SLC7A13* silencing, we added cystine at varying concentrations to the culture medium and analyzed cell numbers using the CCK8 assay. Whereas extra cystines in the medium did not alter the number of cells in the shRNA control group, it rescued the cell number decrease caused by *SLC7A13* silencing in a dose-dependent manner in T-47D cells (Fig. 3F). These findings suggest that elevated SLC7A13 expression in breast cancer cells increases cystine uptake, leading to increased GSH production, reduced ROS accumulation, and attenuated ferroptosis in breast cancer cells.

### Structural determination of SLC7A13-SLC3A1 complex

**Figure 4.**
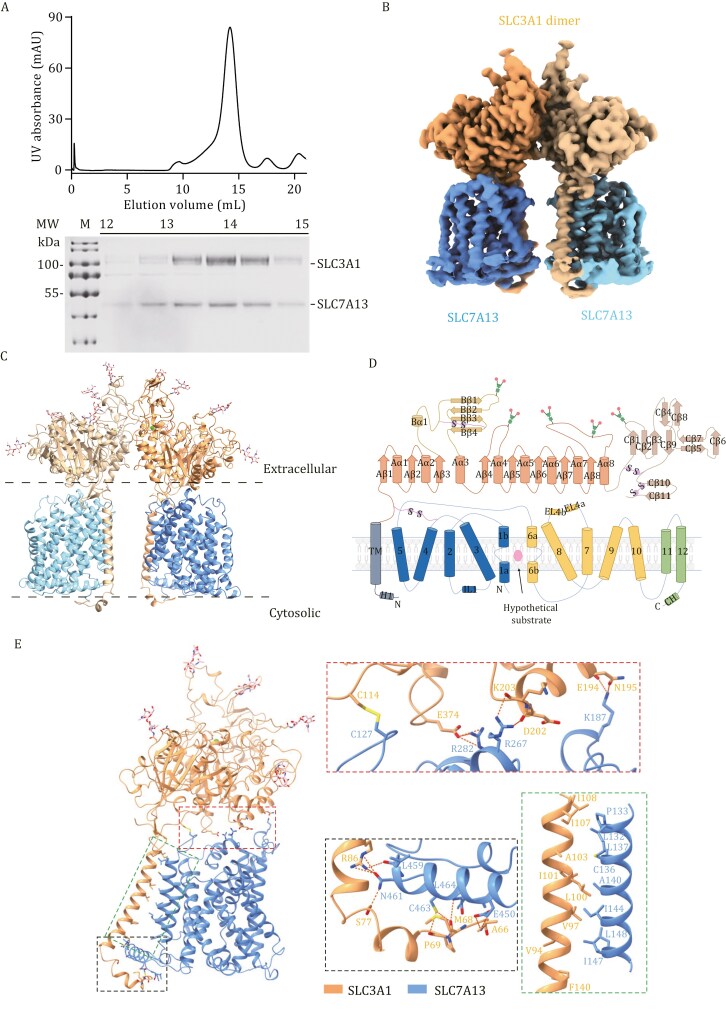
**Structural determination of the SLC7A9/SLC3A1 amino acid transport complex (SLC7A13-SLC3A1).** (A) Gel filtration purification of the SLC7A13-SLC3A1 complex is shown. Fractions from 13‒14 mL on the SDS-PAGE gel were concentrated for cryo-sample preparation. (B) The cryo-EM density map of the SLC7A13-SLC3A1 complex is presented. The SLC7A13-SLC3A1 complex comprises a heterotetrameric assembly of two SLC3A1 and SLC7A13 subunits. (C) A cartoon representation of the atomic model of the SLC7A9-SLC3A1 complex is depicted, with glycosylation moieties shown as sticks. (D) A schematic of SLC3A1 and SLC7A13 secondary structure motifs is provided, with α-helical and β-sheet elements shown as cylinders and arrows, respectively. (E) The atomic model of the SLC7A13-SLC3A1 heterodimer is shown, with a zoomed-in view of the distinct interaction sites between the two subunits. SLC7A13, sky blue; SLC3A1, brown. The red box indicates a detailed view of the interactions at the extracellular interface, with covalent bonds shown as solid yellow lines and hydrogen bonds represented by dashed lines. The green box displays a detailed view of the hydrophobic interactions between the transmembrane helices of SLC7A13 and SLC3A1. The black box presents a detailed view of the SLC7A13-SLC3A1 complex on the intracellular side.

To delve deeper into the molecular mechanism of SLC7A13 in cystine uptake and ferroptosis regulation, we conducted a detailed analysis of the SLC7A13-SLC3A1 complex’s structure. SLC7A13 and SLC3A1 were co-expressed in Expi293 cells, and the complex was purified through affinity purification and size exclusion chromatography (Figs. S3 and 4A). The complex exhibited a single monodisperse peak on size exclusion chromatography (SEC), suggesting high homogeneity (Fig. 4A). The apo structure of the SLC7A13-SLC3A1 complex was determined at an overall resolution of 2.64 Å (Figs. S3–5). Details of cryo-EM sample preparation, data acquisition, and processing are provided in the Materials and Methods section and Fig. S6. The two-dimensional (2D) class average immediately revealed a dimer of the heterodimeric form of the SLC7A13-SLC3A1 complex (Fig. S4A). All sequences of both SLC7A13 and SLC3A1 were resolved in the cryo-EM map, except for several residues of TM6, which showed flexible density (Fig. S5). The overall density map revealed a hetero-tetrameric protein complex composed of two SLC7A13 subunits and two SLC3A1 subunits (Fig. 4B). This architecture is similar to the structures of the human SLC7A9-SLC3A1 (Yan et al., 2020).

As the light chain of a cystine transporter, SLC7A13 comprised 12 transmembrane (TM) helices (Figs. 4C‒E and S4). Resembling other LeuT-fold transporters, it exhibited a canonical LeuT-fold with 10 core TM helices, featuring a 5 + 5 inverted repeat topology, and two additional peripheral TM helices. TM helices 1 and 6 of SLC7A13 were interrupted by a short loop in the middle, denoted as TM1a/1b and TM6a/6b (Fig. 4D). These helices break motifs are conserved and essential for substrate coordination. As the heavy chain of a cystine transporter, SLC3A1 was composed of a small N-terminal cytoplasmic region connected by a single TM to a large extracellular domain (ED) with five glycosylation sites (Fig. 4D). SLC7A13 interacted with SLC3A1 through multiple interfaces among the extracellular side, transmembrane region, and intracellular side, which is quite similar to the SLC7A9-SLC3A1 complex (Fig. 4E).

### Identification of the substrate binding pocket in SLC7A13

**Figure 5.**
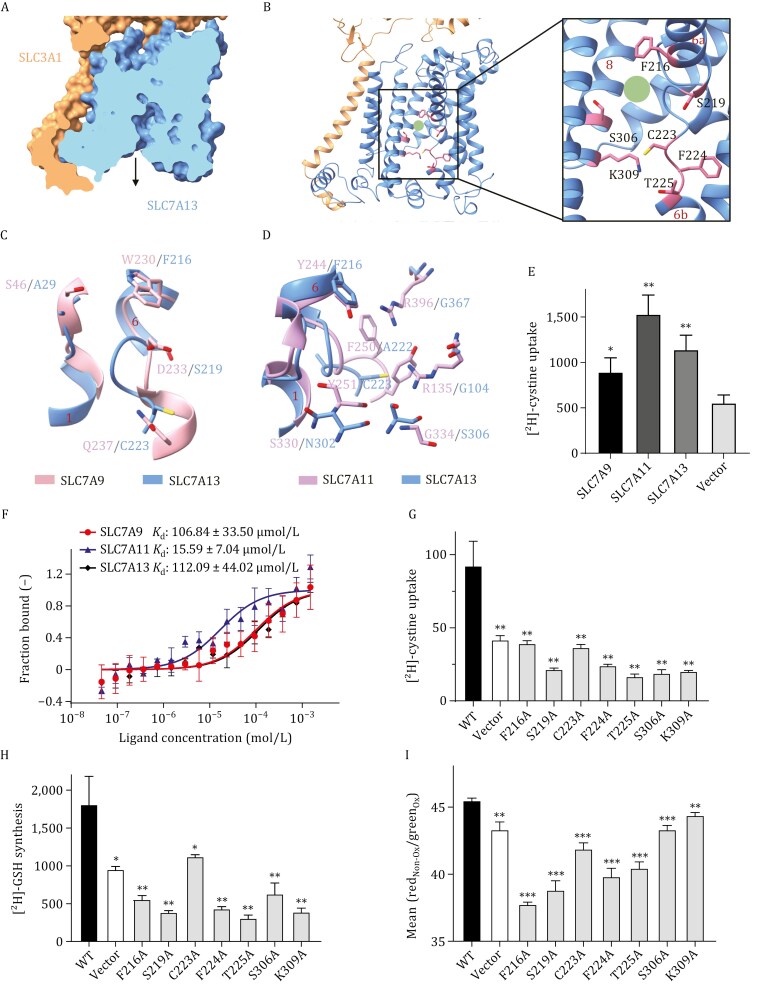
**Cystine binding site and pockets in SLC7A13 and SLC7A9.** (A) SLC7A13 is shown in an inward-open conformation. (B) The intracellular vestibule of the transport pathway is depicted, with key residues involved in transport illustrated as pink sticks. (C) A comparison between SLC7A13 and SLC7A9 (PDB ID: 6LID) is presented. (D) Comparison of AGT1 with SLC7A11 (PDB ID: 7P9V) in the pocket. (E and F) The cystine uptake (E) and binding affinity (F) of SLC7A9-SLC3A1, SLC7A11-SLC3A2, and SLC7A13-SLC3A1 complexes were evaluated. (G‒I) Mutations of residues within the pocket to alanine reduce cystine uptake (G) and GSH biosynthesis (H) in HEK293 cells, as assessed using the [^2^H]-cystine isotope tracking, but has different effects on erastin-induced lipid peroxidation (I), as indicated by the ratio of non-oxidized to oxidized lipids (Y-axis) and analyzed using the flow cytometry. All mutants were compared to the wild type SLC7A13 for *P* values. Data are presented as mean ± SD. *n* = 3. *, *P* < 0.05; **, *P* < 0.01; ***, *P* < 0.001.

The overall structure of the SLC7A13-SLC3A1 complex adopted an inward-facing conformation, closely resembling that of the SLC7A9-SLC3A1 complex (Fig. 5A). Unfortunately, despite the addition of the amino acid substrate, we were unable to obtain structural details of SLC7A13’s substrate binding. Structural analysis revealed that the substrate binding pocket was enclosed by TM6a, TM6b, and TM8 (Fig. 5B).

We aligned the structure of the SLC7A13-SLC3A1 complex to those of the SLC7A9-SLC3A1 (PDB: 6LID) (Yan et al., 2020) (Fig. 5C) and the SLC7A11-SLC3A2 complexes (PDB: 7P9V) (Parker et al., 2021) (Fig. 5D), and identified seven key residues along SLC7A13’s transport path: Phe216, Ser219, Cys223, Phe224, Thr225, Ser306, and Lys309. Three of them, i.e., Phe216, Ser219, and Cys223, are likely critical for substrate binding because they correspond to SLC7A9’s Trp230, Asp233, and Gln237, respectively, which mediate substrate binding via van der Waals interactions and hydrogen bonds (Rodriguez et al., 2021; Yan et al., 2020). Ser219 in SLC7A13’s pocket is likely involved in substrate specificity, as it corresponds to Asp233 in SLC7A9’s pocket, which is essential for recognizing cystine and cationic amino acids (Liu et al., 1999). Similarly, SLC7A13’s Phe216, Cys223, and Ser306 are likely involved in gating, as they correspond to SLC7A11’s Tyr244, Tyr251, and Gly334, respectively, which are critical for gating and cystine entry (Parker et al., 2021).

Among SLC7 members, cystine transport is mediated by SLC7A9, SLC7A11, and SLC7A13. We ectopically expressed the three transporters in HEK293T cells and evaluated their relative uptake rates. We found that the higher a transporter’s expression level was, the higher its cystine uptake rate, with SLC7A11 expressing at a higher level (Fig. S8D) and uptaking more [^2^H]-cystine than SLC7A13 and SLC7A9 (Fig. 5E). We then evaluated their impact on cystine binding affinity using microscale thermophoresis (MST) assays (Fig. 5F). The binding affinities were 106.84 ± 33.50 μmol/L for SLC7A9, 15.59 ± 7.04 μmol/L for SLC7A11, and 112.09 ± 44.02 μmol/L for SLC7A13. SLC7A11 showed significantly higher binding affinity compared to SLC7A13 and SLC7A9. We also compared the transport kinetics (*K*_m_, *V*_max_) for the three transporters (Fig. S8), and the transport rates were as follows: SLC7A9, *V*_max_ = 184.2 ± 30.4 cysteine/cell/min; SLC7A11, *V*_max_ = 278.0 ± 28.9 cysteine/cell/min; SLC7A13, *V*_max_ = 87.04 ± 7.38 cysteine/cell/min. The *V*_max_ of SLC7A11 was higher than that of SLC7A9 and SLC7A13, correlating with their expression levels (Fig. S8D). The *K*_m_ values for SLC7A9, SLC7A11, and SLC7A13 were 276.9 ± 107.6 μmol/L, 67.57 ± 21.95 μmol/L, and 126.5 ± 33.25 μmol/L, respectively, indicating that SLC7A11 binds cystine more strongly than the other two SLC7 members, consistent with the MST assay results.

To further study ligand interactions with SLC7A13, we conducted molecular docking analysis to estimate the binding model of cystine with the SLC7A13 complex. Cystine exhibited mild binding affinity to SLC7A13 (−5.4 kcal/mol) (Fig. S9), and key residues Ser219, Phe224, and Thr225 were also found in the pocket region of the cystine ligand.

As described here, we identified seven residues of SLC7A13, Phe216, Ser219, Cys223, Phe224, Thr225, Ser306, and Lys309, that might be involved in substrate binding. Although we were unable to obtain structural information about SLC7A13’s ligand binding, mutational and functional experiments demonstrated that mutation in any of the seven SLC7A13-specific residues to alanine significantly reduced SLC7A13’s transporter activities, as determined by measuring [^2^H]-cystine uptake and can’t rescue the lipid peroxidation induced by erastin compared to the wild type (WT) in HEK293 cells transfected with *SLC7A13* and *SLC3A1* (Fig. 5G‒I). Therefore, these seven unique residues are critical for SLC7A13’s transport activity.

## Discussion

Tumor cells generate excess oxidative stress due to more active cell proliferation, which requires more cysteine for glutathione biosynthesis to detoxify lipid peroxides. Meanwhile, cysteine synthesis is either inactive or downregulated during tumorigenesis, so cystine uptake becomes more active in cancer cells as a compensatory mechanism (McBean, 2002; Yoon et al., 2023). Depletion of cystine causes the accumulation of ROS and subsequent ferroptosis. Cancer cells overexpressing a cystine transporter, such as SLC7A11 in basal breast cancer (Nath et al., 2024) and SLC7A13 in luminal breast cancer (Fig. 1), are likely to become more susceptible to ferroptosis when cystine is depleted. Blocking cystine uptake has thus been considered a therapeutic strategy in cancer treatment (McBean, 2002; Yoon et al., 2023). Our findings in this study suggest that SLC7A13 is a potential therapeutic target for treating luminal breast cancer.

Firstly, the *SLC7A13* gene is amplified in about 20% and overexpressed in the majority of breast cancers (Fig. 1). More importantly, *SLC7A13*’s amplification or overexpression is significantly associated with higher tumor grades and worse patient survival in breast cancer (Fig. 1). A previous study demonstrated that elevated SLC7A13 levels were positively correlated with higher tumor stages in breast cancer tumor (Yan et al., 2022a). Functionally, elevated *SLC7A13* expression promotes the proliferation or survival of breast cancer cells, as demonstrated by various assays that measure cell survival and/or proliferation in two breast cancer cell lines with *SLC7A13* overexpression (Fig. 2).

SLC7A13 has been previously identified as the second partner of SLC3A1, and the SLC3A1-SLC7A13 complex has been confirmed to transport cystine (Nagamori et al., 2016). We demonstrated that silencing *SLC7A13* dramatically reduced cystine uptake and GSH biosynthesis in breast cancer cells, leading to increased ROS accumulation (Fig. 3). These effects of *SLC7A13* silencing could be rescued by supplementing the culture medium with more cystine (Fig. 3). It is highly likely that cysteine transporters exhibit functional redundancy. However, breast cancer cells overexpressing SLC7A13 could develop an addiction to this transporter, and targeting SLC7A13 could still kill cancer cells by ferroptosis even if SLC7A9 or SLC7A11 is normally expressed in the same breast cancer cells (Figs. 2 and 3). Therefore, it is less likely that functional redundancy among the transporters could compromise the therapeutic value of targeting SLC7A13 or SLC7A11. Taken together with the well-documented role of more cystine uptake in reducing lower ROS levels and ferroptosis, we conclude that elevated *SLC7A13* expression plays a promoting role in breast cancer.

*SLC3A1*’s expression levels are also higher in breast cancer; higher *SLC3A1* levels are also correlated with higher clinical stages and worse patient survival; and ectopic expression of *SLC3A1* enhances tumor growth (Jiang et al., 2017). In three of the seven breast cancer cell lines tested, SLC3A1 protein level was relatively higher, including ZR-75-30, T-47D, and MCF-7 (Fig. 2A). Interestingly, the 2 cell lines with SLC7A13 overexpression also overexpressed SLC3A1, including T-47D and MCF-7 (Fig. 2A), suggesting a correlation between SLC3A1 and SLC7A13 protein levels. Loss of SLC3A1 in mouse kidneys eliminates SLC7A13’s expression in the renal apical membrane (Nagamori et al., 2016). It is thus possible that elevated *SLC3A1* expression could cause the elevation of *SLC7A13* expression in breast cancers without *SLC7A13* amplification (Fig. 1). Therefore, overexpression of the SLC3A1-SLC7A13 transporter complex is frequent in breast cancer, and the promotion of breast cancer by increased cystine uptake and the overcoming of ferroptosis could be rather prevalent.

The L-type amino acid transporter superfamily includes SLC7A5-SLC7A13 and SLC7A15. Three members of this superfamily have been identified as cystine transporters, including SLC7A9, SLC7A11, and SLC7A13 (Closs et al., 2006; Verrey et al., 2004). Of the three cysteine transporters, SLC7A11 (also known as xCT) also plays a role in human cancers, including breast cancer, as it has emerged as a potential therapeutic target for cancer treatment due to its overexpression in multiple human cancers. Its overexpression promotes tumor growth in part by suppressing ferroptosis (Koppula et al., 2021). In breast cancer, particularly, SLC7A11 has been identified as a common therapeutic target for triple-negative breast cancer, as its expression is significantly elevated in these tumors, and the elevation is significantly associated with higher tumor grades (Nath et al., 2024; Timmerman et al., 2013).

Therefore, while both SLC7A13 and SLC7A11 play crucial roles in breast cancer, they modulate different subtypes of the disease. Whereas SLC7A11 overexpression predominantly impacts the basal or triple-negative subgroup of tumors (Nath et al., 2024; Timmerman et al., 2013), SLC7A13 overexpression is primarily involved in the luminal subgroup (Figs. 1 and 2). In addition, our findings in the current study further support the concept that cystine transporters are valid therapeutic targets in cancer treatment.

SLC7A9 and SLC7A13 share high sequence similarity, and both bind to SLC3A1 as their common partner, while SLC7A11 forms a transport complex with the heavy chain SLC3A2. SLC3A1 mediates the dimerization of HATs, which consist of two light chain subunits and two heavy chain subunits of SLC3. System xc^−^ comprises the light chain SLC7A11 and the heavy chain SLC3A2. The differences in cystine recognition among SLC7A13, SLC7A9, and SLC7A11 may be attributed to the variations in their heavy chain associations and HAT aggregation modes, as well as in cystine ligand binding mechanisms.

As described in the Results section, we solved the cryo-EM structure of the SLC7A13-SLC3A1 transporter complex, in which the active pocket and residues unique to this complex were identified. We also compared the differences of three cystine transporters, SLC7A13, SLC7A9, and SLC7A11. These findings would facilitate an understanding of cystine transport and the mechanisms by which the dynamic interactions between the SLC7A13-SLC3A1 transporter and its ligands, including the binding sites, occur. Our results would also facilitate the design of new drugs with higher activities and specificities in targeting cystine addiction as a metabolic vulnerability in cancer treatment.

In summary, we demonstrated that the recently identified SLC7A13-SLC3A1 cystine transporter is overexpressed in human breast cancers, and this overexpression is significantly associated with higher tumor grades and poorer patient survival. Functionally, elevated *SLC7A13* expression promotes the proliferation and survival of breast cancer cells. *SLC7A13* silencing dramatically reduced cystine uptake and GSH biosynthesis, while increasing ROS levels; these effects could be reversed by cystine supplementation. Additionally, we resolved the cryo-EM structure of the SLC7A13-SLC3A1 complex and identified its substrate binding pocket. These findings not only highlight SLC7A13 as a potential therapeutic target in breast cancer but also provide structural insights into its biochemical mechanisms, paving the way for the design of more effective and specific drugs targeting cystine addiction in breast cancer treatment.

## Supplementary data

Supplementary data is available at *Protein & Cell* online https://doi.org/10.1093/procel/pwaf076.

pwaf076_Supplementary_Figures_S1-S9

## Data Availability

Atomic coordinate and cryo-EM density map of the SLC3A1- SLC7A13 complex in the apo state (PDB: 8WK6; whole map: EMD-37596) have been deposited to the Protein Data Bank (rcsb.org) and the Electron Microscopy Data Bank (https://www.ebi.ac.uk/pdbe/emdb/), respectively. All other data will be made available upon request. Source data are provided in this paper. Correspondence and material requests should be addressed to Renhong Yan (yanrh@sustech.edu.cn) and Jin-Tang Dong (dongjt@sustech.edu.cn)".

## Code availability

This paper does not report original code.
