# Supplementary material for: Oncogenic role of the SLC7A13-SLC3A1 cystine transporter in human luminal breast cancer and its cryo-EM structure
Source: Protein Cell. 2025 Sep 6;17(2):145–56. doi: 10.1093/procel/pwaf076 (PMC12959773; doi:10.1093/procel/pwaf076)
Supplement: pwaf076_Supplementary_Figures_S1-S9 [file pwaf076_supplementary_figures_s1-s9.pdf]

Supplementary Materials for

Oncogenic role of the SLC7A13-SLC3A1 cystine transporter in human luminal breast cancer and its cryo-EM structure

Jing Dong[1]†, Tianhao Shi[2]†, Bingbiao Lin[1], Xuetong Liu[1], Waner Wei[1], Zichi Geng[1], Mingcheng Liu[1], Renhong Yan[2]*, Jin-Tang Dong[1]*

Corresponding author: dongjt@sustech.edu.cn (J.T.D.); yanrh@sustech.edu.cn (R.Y.)

The PDF file includes the following sections:

Materials and Methods
Figures. S1 to S11
Table S1
References

Materials and Methods

Bioinformatic and statistical analyses

The data on patient survival, tumor grade, and *SLC7A13*'s copy number alterations were retrieved from the Molecular Taxonomy of Breast Cancer International Consortium (METABRIC) database via cBioPortal (Pereira et al., 2016, Rueda et al., 2019, Curtis et al., 2012). *SLC7A13* alterations include amplifications and homozygous deletion (homdel). Kaplan-Meier analysis was used to evaluate the associations between *SLC7A13* amplifications and overall survival or relapse-free survival. A log-rank test was used to derive the *p*-values. Univariate Cox regression analysis was applied to calculate the hazard ratios. Fisher's exact test was conducted to assess the associations of *SLC7A13* amplifications with tumor grades.
We also compared *SLC7A13*'s mRNA expression levels in breast cancer and normal breast tissues using data from the TCGA database. To associate *SLC7A13*'s expression levels with patients' overall survival, we used the GSE9893 dataset (Chanrion et al., 2008) containing survival information and performed the Kaplan-Meier analysis.
Breast cancers in the TCGA database were divided into *SLC7A13*-high and low groups based on *SLC7A13*'s median expression level. Differentially expressed genes between the two groups were then identified and subjected to the KEGG enrichment analysis to identify SLC7A13-associated signaling pathways. The same analyses were also applied to *SLC7A11*.
The estrogen activity in the TCGA breast cancers was inferred using the PROGENy package with default parameters. Pearson correlation analysis was then conducted to determine the correlation between the estrogen activity and SLC7A13's or *SLC7A11*'s mRNA levels.

Cell culture

All cell lines were procured from the American Type Culture Collection (ATCC; Manassas, VA) and were cultured at 37°C in a humidified incubator with 5% CO_2. HEK293, HeLa, and MCF-7 cell lines were cultured in the DMEM medium (VivaCell, Shanghai, China) supplemented with 10% fetal bovine serum (FBS, WISENT, Nanjing, China). The remaining cell lines were cultured in the RPMI 1640 medium (VivaCell) supplemented with 10% FBS.

Estrogen-responsive expression of *SLC7A13* in ER-positive breast cancers

T-47D cells on 6-well plates were cultured in phenol red-free medium supplemented with 5% charcoal dextran-stripped FBS for 3 days. Then they were inoculated with the same medium containing various concentrations of estradiol (E_2) for 12 hours, with ethanol as a control. Real-time qPCR was then performed to detect gene expression (see below), with the ER-responsive *CATD* gene as a positive control.

Real-time qPCR

Total RNA was extracted using the Total RNA Extraction Kit (Promega, Beijing, China) according to the manufacturer's instructions. Complementary DNA (cDNA) was synthesized using the HiScript® III RT Super Mix for qPCR (Vazyme, Nanjing, China). Quantitative real-time PCR was performed using 2×SYBR qPCR Master Mix (MIKX, Shenzhen, China) in a qTOWER PCR system (Analytik Jena AG, Germany). Primer sequences used in the study are as follows: *SLC7A13*, 5'-CTCGTGGTGTGAAAGAAGTGAC-3' (forward), 5'-GGAACACTACTCCAGTTAGGGAA-3' (reverse); *SLC7A11*, 5'-TTTCAAGGTGCCACTGTTCATCC-3' (forward), 5'-

ACGAAGCCAATCCCTGTACTAAATG -3' (reverse); *cathepsin D* (*CATD*), 5'-ACTGCTGGACATCGCTTGCT-3' (forward), 5'- TGTCAAACGAGGTACCATTCTTC-3' (reverse); *GAPDH*, 5'-GGTGGTCTCCTCTGACTTCAACA-3' (forward), 5'-GTTGCTGTAGCCAAATTCGTTGT-3' (reverse).

Knockdown of SLC7A13 in breast cancer cells

For the establishment of a stable cell population expressing shRNA against SLC*SLC7A13*, two distinct *SLC7A13* shRNAs were used, with the target sequences of GCTTGGTTGTGATACCATTGG and GCATTATGTCTACGTGCTTCT. The control shRNA had the sequence of GGTTCTCCGAACGTGTCACGT. Complementary single strands of shRNA were synthesized, annealed, and cloned into the pLKO.1-puro vector as described by the provider. These constructs were co-transfected into HEK293T cells with the lentivirus helper plasmids psPAX2 and pMD2.G. Forty-eight hours later, media from cells were collected, filtered, and added to MCF-7 and T-47D breast cancer cells. The culture medium was replaced by a selection medium containing 2 μg/ml puromycin, and cells were selected for at least 7 days.

Western blotting

Antibodies used in this study included SLC7A13 (1:500 dilution, Abcepta, # AP10029c, Suzhou, China), SLC3A1 (1:1000 dilution; Proteintech, catalog #16343, Wuhan, China), GPX4 (1:1000 dilution; ABclonal, catalog #A11243, Wuhan, China), and Na+/K+ ATPase (1:1000 dilution, Abbkine, catalog #ABP51894, Wuhan, China). Cell lysates were separated using SDS-PAGE for western blotting, and proteins were transferred onto polyvinylidene fluoride membranes (Millipore, Billerica, MA) following standard protocols. Membranes were blocked with 5% nonfat milk, incubated with the primary antibody overnight at 4 °C, and incubated with a horseradish peroxidase-conjugated secondary antibody for 1 hour at room temperature. Protein signals were detected using a luminescent image analyzer (Champchemi 610plus, Beijing, China) after applying the Western Bright ECL reagents (Advansta, Menlo Park, CA).

Sulforhodamine B (SRB) and CCK8 assays

Cells were seeded onto 24-well plates at 3×10^4 cells/well. At 1, 2, 4, and 6 days after seeding, cells were fixed in 10% trichloroacetic acid (TCA), stained with 0.4% SRB (Sigma-Aldrich, Taufkirchen, Germany), and rinsed with 1% acetic acid. The absorbance at 490 nM was measured using a spectrometer.

For the CCK8 assay, cells were seeded onto 96-well plates at 5,000 cells/well. After the cells were attached to the plastic surface, the culture medium was replaced with that containing various concentrations of cysteine, and the culture was continued. Forty-eight hours later, cells were incubated in the medium containing 10% (v/v) CCK8 solution (YEASEN, Shanghai, China) for about 1h. Cell samples were then measured for absorbance at 450 nm using a spectrometer.

Colony formation assay and anchorage-independent growth analysis

For the colony formation assay, 1,000 cells were seeded onto 6-well plates and cultured for 15 days with the medium replaced every 3 days. Cells were then fixed using 4% polyformaldehyde and stained with 0.05% crystal violet. For the anchorage-independent growth analysis, a base layer of soft agar was prepared by adding 1.5 mL of medium containing 0.6% soft agar (Lonza, Rockland, ME, USA) to each well of a 6-well plate. To each well, 5,000 cells in 1.5 mL of

medium containing 0.35% agar were added, followed by 1 mL of medium after the agar had hardened. Cells were incubated for approximately two weeks until colonies were visible. Images of colonies were captured using an inverted microscope (Eclipse TI2, Nikon), and their numbers were determined using the ImageJ software.

Mass spectrometric analysis with isotope tracking

To determine the effect of SLC7A13 on cystine uptake and GSH biosynthesis, T-47D breast cancer cells were seeded onto 6-well plates and *SLC7A13* shRNAs as described above. To determine the effects of *SLC7A13* mutations on cystine uptake and GSH biosynthesis, HEK293 cells were seeded onto 6-well plates and transfected with the pCAG-FLAG-SLC7A13 described above and its mutants of F216A, S219A, C223A, F224A, T225A, S306A, and K309A in the presence of the pCAG-TS-SLC3A1 plasmid described above. These SLC7A13 mutants were generated from the pCAG-FLAG-SLC7A13 plasmid using the overlap extension PCR approach. After 48 hours of transfection, cells were cultured in a medium containing 300 μM [^{2}H]-cystine (3,3,3',3'-D4, Cambridge Isotope Laboratories, Boston, MA) for 12 hours. Subsequently, cells were washed 3 times with ice-cold PBS and harvested in 80% methanol containing 25 mM N-ethylmaleimide (Alfa Aesar, Ward Hill, MA, USA) and 0.04 μM L-4-chlorophenylalanine (Sigma Aldrich, St Louis, MO, USA). Cells were broken by repeated freezing in liquid nitrogen and thawing at room temperature, and the supernatant was collected by centrifugation at 12,000 rpm for 10 min at 4°C.

The *Km* and *Vmax* values for the amino acid substrates were determined using Michaelis-Menten kinetics, based on the uptake of [^{2}H]-cystine mediated by SLC7A9, SLC7A11, and SLC7A13. The measurements were conducted at substrate concentrations of 5, 10, 30, 50, 100, 300, 500, and 1000 μM.

Using a Q Exactive mass spectrometer coupled with an UltiMate 3000 liquid chromatography system (Thermo-Fisher), 10 μL supernatant was injected into a Hypersil GOLDTM HILIC column (2.1 ×100 mm, 1.9 μm, Thermo Scientific) and run through the column at a flow rate of 0.3 ml/min, along with data acquisition. Mobile phase A consisted of 0.1% formic acid in H_2O, whereas mobile phase B consisted of 0.1% formic acid in acetonitrile. The mass spectrometer was operated in positive mode with a spray voltage of 3.5 kV. Full scan mass spectrometry data were recorded from the m/z range of 80−600.

Detection of lipid ROS

Cells were seeded onto 6-well plates. Erastin treatment was 24 hours with 20 μM for T-47D cells and 50 μM for HEK293T cells. Cells were then incubated with the medium containing 5 μM C11-BODIPY 581/591 dye (Invitrogen, CA, USA) at 37°C for 30 minutes. After rinsing with PBS twice, cells were analyzed at approximately 300 events per second on a flow cytometer with the green (488 nm laser excitation and fluorescence emission collected at 530/30 nm) and red channels (561 nm laser excitation and fluorescence emission collected at 610/20 nm). The BD FACSAria™ III system (BD Bioscience, Franklin Lakes, USA) was used to detect fluorescence signals, and data were analyzed using the FlowJo software (BD Biosciences). The data are presented as the ratio of red/green fluorescence intensities.

Protein expression and purification

Full-length cDNA of *SLC7A13* (accession number: NM_138817.3) was purchased from WZ Biosciences (Jinan, China) and cloned into the pCAG vector with an N-terminal FLAG tag as

previously described (Yan et al., 2020). The resultant plasmid was named pCAG-FLAG-SLC7A13. The *SLC3A1* expression construct in the same vector with an N-terminal TwinStrep tag, pCAG-TS-SLC3A1, was from our previous study (Yan et al., 2020). HEK293F cells were cultured in the SMM 293 T-II medium (Sino Biological Inc.) at 37°C and 5% CO_2 in a shaker incubator with a rotation speed of 130 rpm in a Multitron-Pro shaker until the cell density reached 2.3-2.8 × 10^6/ml. For transfection, 50 ml PBS containing 3 mg of polyethyleneimine (Polysciences), 0.75 mg of the *SLC3A1* plasmid, and 0.75 mg of the *SLC7A13* plasmid were incubated at room temperature for 15 min and then added to 1 liter of cell culture. Cells were harvested 60 hours after transfection.

For the purification of the SLC3A1-SLC7A13 complex, cells were collected by centrifugation at 3800g for 10 minutes and resuspended in a buffer containing 25 mM HEPES (pH 7.5), 150 mM NaCl, and 3 protease inhibitors (aprotinin 0.2μM; pepstatin, 1μM; and leupeptin, 10μM, all from Amresco (Solon, OH, USA). Cells were solubilized at 4°C for 2 hours with 1% lauryl maltose neopentyl glycol (LMNG) (Anatrace, Maumee, OH, USA) and 0.1% cholesteryl hemisuccinate, CHEMS (Avanti Polar Lipids, Alabaster, AL, USA). Cell debris was removed by centrifugation at 18,700g for 45 minutes at 4°C, and the supernatant was collected and loaded onto a column filled with the Anti-DYKDDDDK IP Resin (Genscript, Nanjing, China). The resin was then washed with the wash buffer (25 mM HEPES, pH 7.5, 150 mM NaCl, and 0.06% digitonin (Biosynth, Naperville, USA), w/v), and the protein was then eluted with the wash buffer containing FLAG peptide at 0.2 mg/ml. The eluent was then loaded onto a chromatography column filled with Strep-resin (IBA Life Sciences). After washing, the protein complex was eluted from the column with the wash buffer containing 50 mM biotin. Subsequently, size exclusion chromatography was carried out using the Superose 6 Increase 10/300 GL column (GE Healthcare, Milwaukee, WI, USA) and a buffer containing 25 mM HEPES (pH 7.5), 150 mM NaCl, and 0.06% digitonin. The peak fractions were collected and concentrated for cryo-EM analysis.

Cryo-EM sample preparation and data acquisition

The purified SLC3A1-SLC7A13 protein complex was concentrated at 6 mg/ml. A 3 μL protein complex aliquot was then applied to a membrane with glow-discharged holey carbon grids (Quantifoil Au R1.2/1.3, Jena, Germany), which was then blotted for 3.5 seconds and then flash-frozen in liquid ethane and further cooled in liquid nitrogen using a Vitrobot (Mark IV, Thermo-Fisher Scientific). The prepared sample grids were transferred to a Titan Krios operating at 300 kV and equipped with a Gatan K3 Summit detector and a GIF Quantum energy filter (Thermo-Fisher). Movie stacks were automatically collected using the EPU software, with a slit width of 20 eV on the energy filter and a defocus range from -1.3 μm to -1.8 μm in super-resolution mode at a nominal magnification of 81,000x. Each stack was exposed for 2.56 seconds with an exposure time of 0.08 seconds per frame, resulting in 32 frames per stack. The total dose rate was approximately 50 e^-/Å2 for each stack. The stacks were motion corrected with the EMshark and binned 2-fold programs, resulting in a pixel size of 1.095 Å/pixel. Concurrently, dose weighting was performed. The defocus values were estimated with Gctf.

Cryo-EM Data processing

The structure of SLC3A1-SLC7A13 was determined using the CryoSPARC package. After importing the motion-corrected datasets, filtering micrographs were determined using the Manually Curate Exposures program. All selected micrographs underwent patch CTF estimation

and particle selection using the Blob picker. Following several rounds of 2D classification, appropriate 2D templates were chosen, and the Template picker was utilized to select more suitable particles. Subsequently, multiple rounds of 2D classification were carried out to narrow the particle selection further. Selected particles then underwent several rounds of heterogeneous refinement with C1 symmetry. A 3D model lacking the transmembrane regions was generated using ChimeraX and continued with the classification using C2 symmetry. Non-uniform refinement was performed on these particles, resulting in the generation of high-resolution reconstructions. The resolution was estimated using the gold-standard Fourier shell correlation with a threshold of 0.143, combined with the high-resolution noise substitution method.

Model building and structural refinement

Model construction was executed using Phenix and Coot. The initial structure was predicted using the AlphaFold program, which was subsequently aligned with the corresponding electron density maps using ChimeraX, and the structures of SLC7A13 and SLC3A2 were integrated. Subsequent refinement was realized using the real-space refinement function of Phenix. Additional improvements were made through manual adjustments in Coot. Detailed refinement statistics are available in Table S1. Figures illustrating structural features were created using ChimeraX.

Molecular docking

Docking simulation with the software AutoDock Vina (version 1.2.0) was used to investigate the interaction of proteins and ligands, according to the following procedures: (i) Preparation of template structures of remodeled SLC7A13-SLC3A1 complex. The missing polar hydrogen was added using the AutoDockTool software. Subsequently, it was saved in a dockable PDBQT format for the following virtual work. (ii) Docking ligand preparation. The structure data file format of small molecules (cystine) was obtained from the PubChem website (https://pubchem.ncbi.nlm.nih.gov) and minimized energy using the MM2 algorithm. The PDBQT chemical formats of cystine were created by the AutoDock Tool software. (iii) Set docking grid box. The Auto-DockTool software was used to build up docking grid box parameter files of the SLC7A13-SLC3A1 complex, respectively, based on the active pocket. (iv) Molecular docking was performed by AutoDock Vina, and the final results were processed with PyMOL (version 1.4.1). A substrate pocket, which is 5 Å from cystine, is depicted.

Microscale Thermophoresis (MST)

Full-length coding sequences of SLC7A9, SLC7A11, and SLC7A13 were cloned into a GFP-pCAG vector. These plasmids were then transformed with SLC3A1 or SLC3A2 into HEK-293F cells for protein expression. The subsequent extraction and purification of the protein followed the same procedures as described above. MST experiments were conducted using a Monolith NT.115Pico optics system (NanoTemper Technologies). The GFP protein was used at a final concentration of 100 nM. Cystine was initially dissolved in 1M HCl to create a 300 mM stock solution. Before use, the cystine was diluted to 3 mM with a protein buffer at a pH of about 7.0. A two-fold serial dilution series of the cystine in protein buffer was prepared. For each assay point, 10 μL of a specific dilution was mixed with 10 μL of the GFP-protein solution. The prepared samples were loaded into hydrophilic capillaries (NanoTemper Technologies) for MST measurement. The binding affinity assay was independently replicated at least three times. Data

obtained from these experiments were combined and analyzed using the MO Affinity Analysis software (Version 2.3).

Detection of cellular localizations of SLC7A13 and SLC3A1

Cellular localization of SLC7A13 and SLC3A1 was investigated in HeLa cells. The *SLC3A1* and *SLC7A13* cDNAs were cloned into the pmCherry vector (gift of Dr. Mingjie Zhang of SUSTech) and pEGFP vector (Sun et al., 2014), respectively. The resultant plasmids were named pmCherry-SLC3A1 and pEGFP-SLC7A13. Cells were seeded onto coverslips and transfected with pmCherry-SLC3A1 and pEGFP-SLC7A13 at a 1:1 molar ratio. After 48 hours of culture, cells were stained with DAPI. Images were captured using the LSM980 confocal microscope (Zeiss).

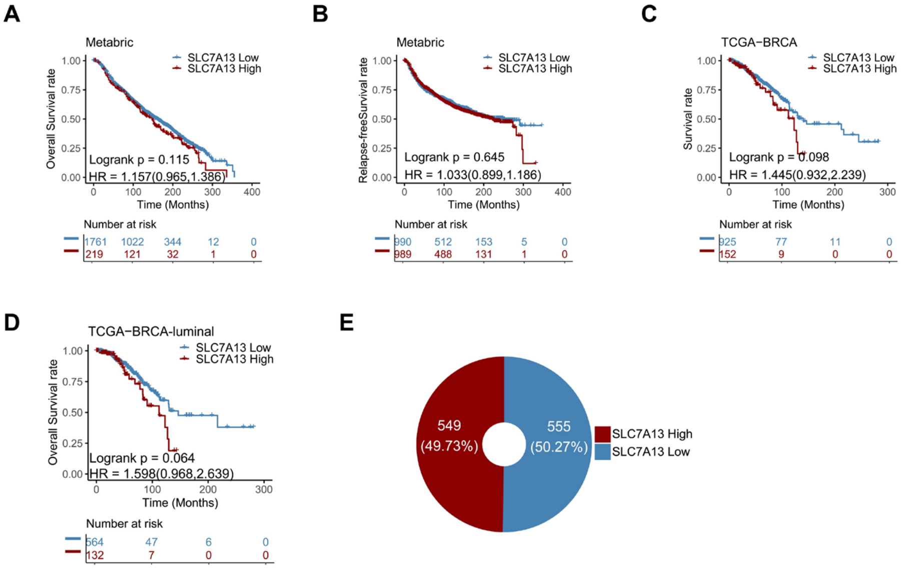

Fig. S1. Lack of association between *SLC7A13* expression and patient survival in the METABRIC and TCGA breast cancer datasets. The Kaplan-Meier analysis was used to determine the association. **(A)** The METABRIC cohort for overall survival. **(B)** The METABRIC cohort for refractory-free survival. **(C)** The TCGA dataset for overall survival. **(D)** Higher SLC7A13 levels correlated with the overall survival of patients with luminal breast cancers, as analyzed using the Kaplan-Meier analysis and the TCGA breast cancer dataset. **(E)** Distribution of breast cancers with higher and lower *SLC7A13* expression levels, where the *SLC7A13* level was higher than (*SLC7A13*-high), or equal to/lower than (*SLC7A13*-low), twice the average *SLC7A13* level in normal breast tissues.

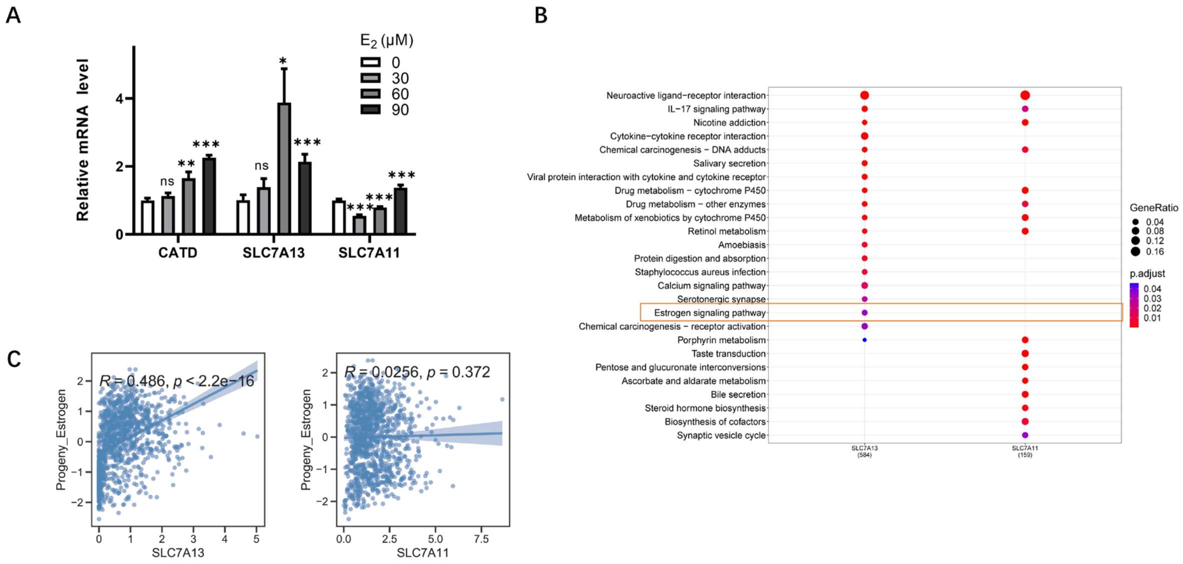

Fig. S2. *SLC7A13* expression responds to the estrogen/ER signaling activity. (A) Estrogen (E_2) induces *SLC7A13* expression in a dose-dependent manner. **(B)** Enrichment of the estrogen signaling pathway in *SLC7A13*-associated genes but not in those of *SLC7A11*, as determined using the KEGG analysis using the TCGA database. Pathways with statistical significance ($p < 0.05$) were listed. Dot sizes represent the proportion of genes involved in a pathway, and dot colors represent p-values. **(C)** Pearson correlation analysis of the estrogen activity and mRNA levels of *SLC7A13* or *SLC7A11*. Data are presented as mean ± SD. n = 3. *, P <0.05; **, P < 0.01; ***, P < 0.001.

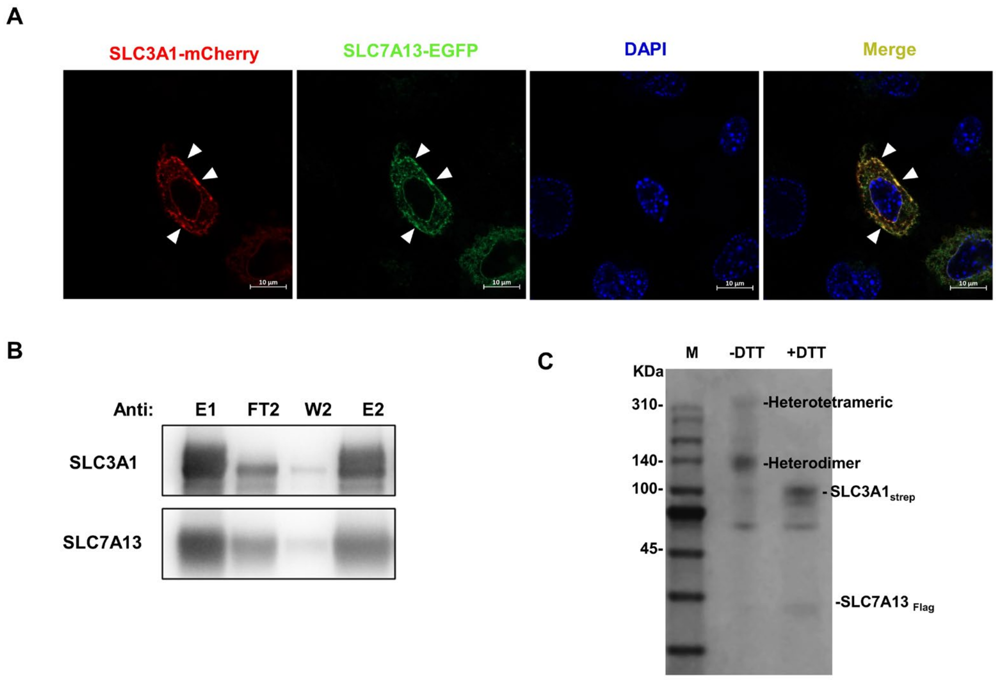

Fig. S3. Biochemical identification of the SLC7A13-SLC3A1 (AGT1–rBAT) Complex. (A) Overexpression of SLC7A13-GFP and SLC3A1-mCherry in HEK293 cells indicates that SLC7A13 and SLC3A1 form complexes on the cell membrane. **(B)** Detection of SLC7A13 and SLC3A1 after a two-step affinity purification of the complex. E1, eluent of protein purified by M2 affinity; FT2, protein detection after flowing through strep column; W2, bleach solution for strep columns; E_2, strep column eluent. **(C)** SDS-PAGE analysis of SLC7A13–SLC3A1. The purified complex was subjected to non-reducing (-DTT) and reducing SDS-PAGE (+DTT).

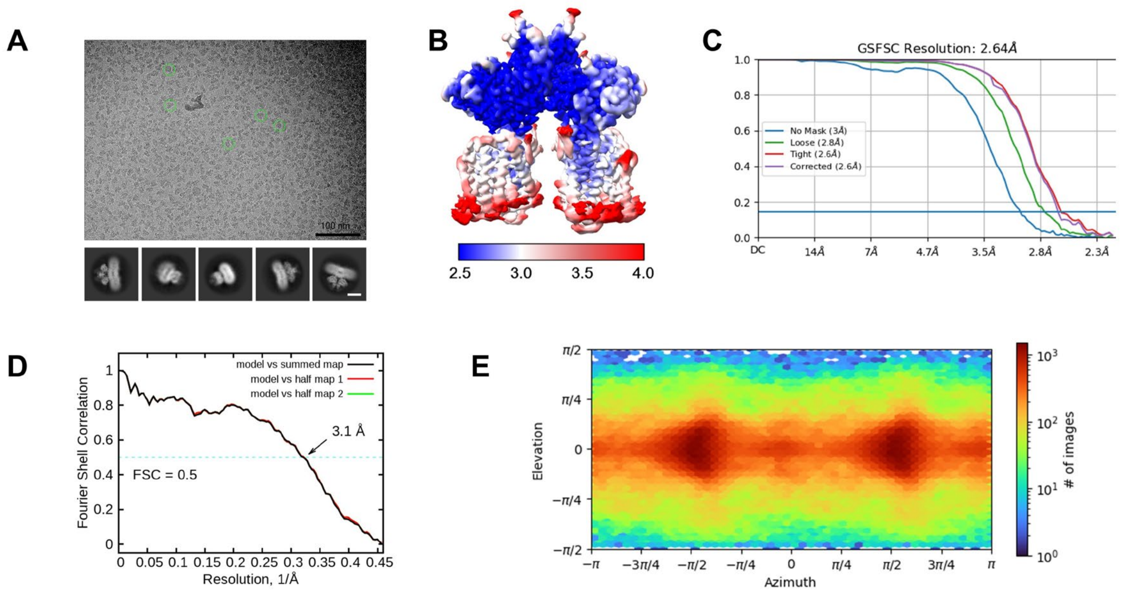

Fig. S4. Cryo-EM analysis of the SLC7A13-SLC3A1 complex. (A) Representative cryo-EM micrograph and 2D class averages of cryo-EM particle images. The scale bar in the 2D class averages 10 nm. **(B)** Local resolution maps for the 3D EM reconstruction of the overall structure of the SLC7A13- SLC3A1. **(C)** Gold standard FSC curve of the cryoSPARC 3D reconstruction of the whole map. **(D)** FSC curve of the refined model of the SLC7A13- SLC3A1 versus the overall map that it is refined against (black); of the model refined against the first half map versus the same map (red); and of the model refined against the first half map versus the second half map (green). The slight difference between the red and green curves indicates that the refinement of the atomic coordinates did not suffer from overfitting. **(E)** Euler angle distribution of the SLC7A13- SLC3A1 complex.

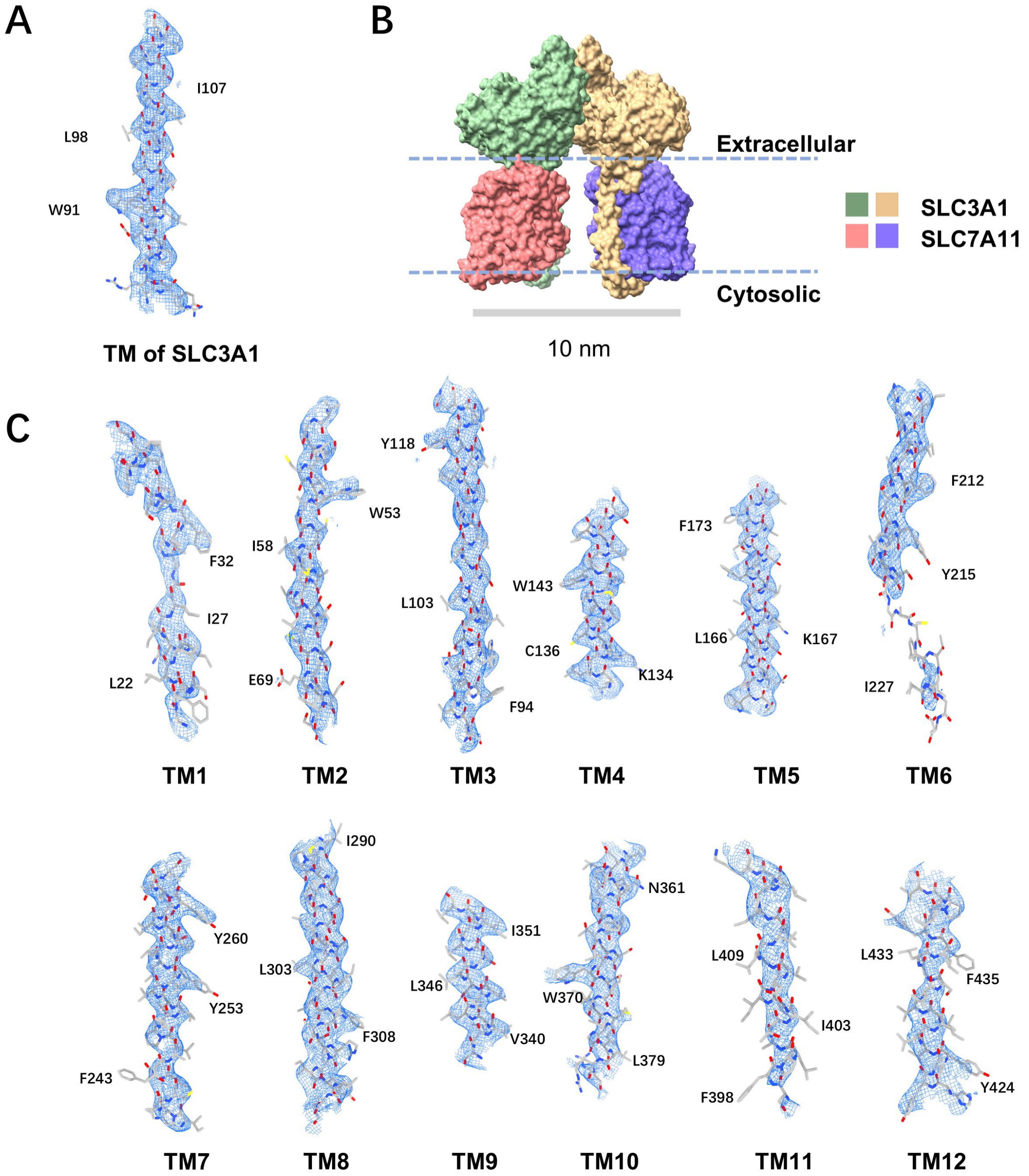

Fig. S5. Representative cryo-EM density maps of SLC7A13 and SLC3A1. (A) Cryo-EM density map of the transmembrane domain of SLC3A1 is shown at a threshold of 5 σ. **(B)** The molecular surface of the SLC7A13- SLC3A1 complex. **(C)** The cryo-EM density map of the transmembrane domain of SLC7A13 is shown at a threshold of 5 σ.

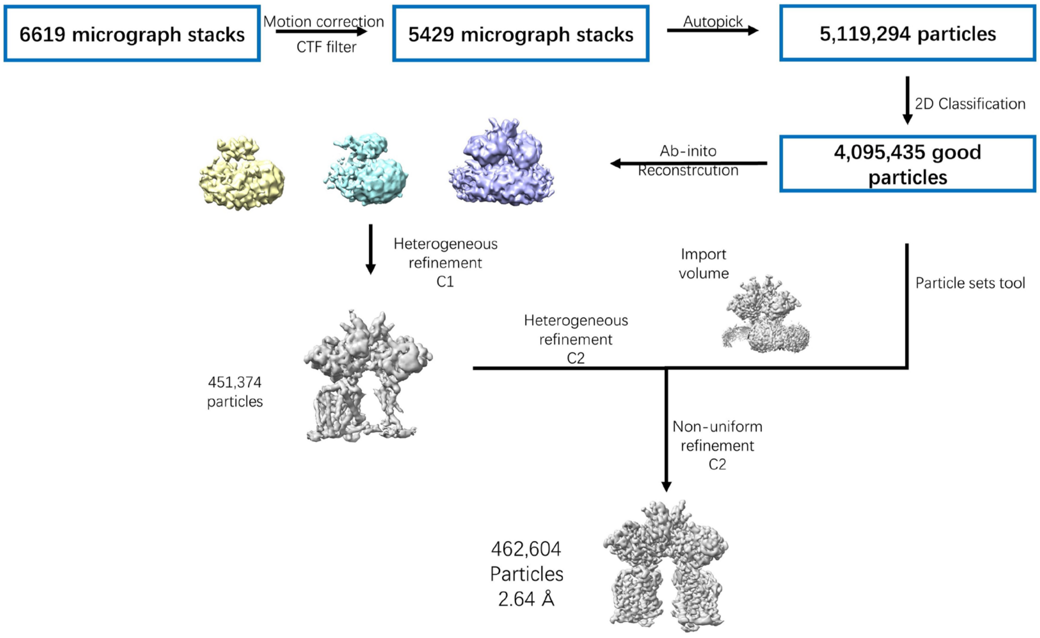

Fig. S6. Cryo-EM data processing workflow for the SLC7A13- SLC3A1 complex. Flowchart for cryo-electron microscopy data processing. For detailed information, please refer to the 'Data Processing' section in the Methods.

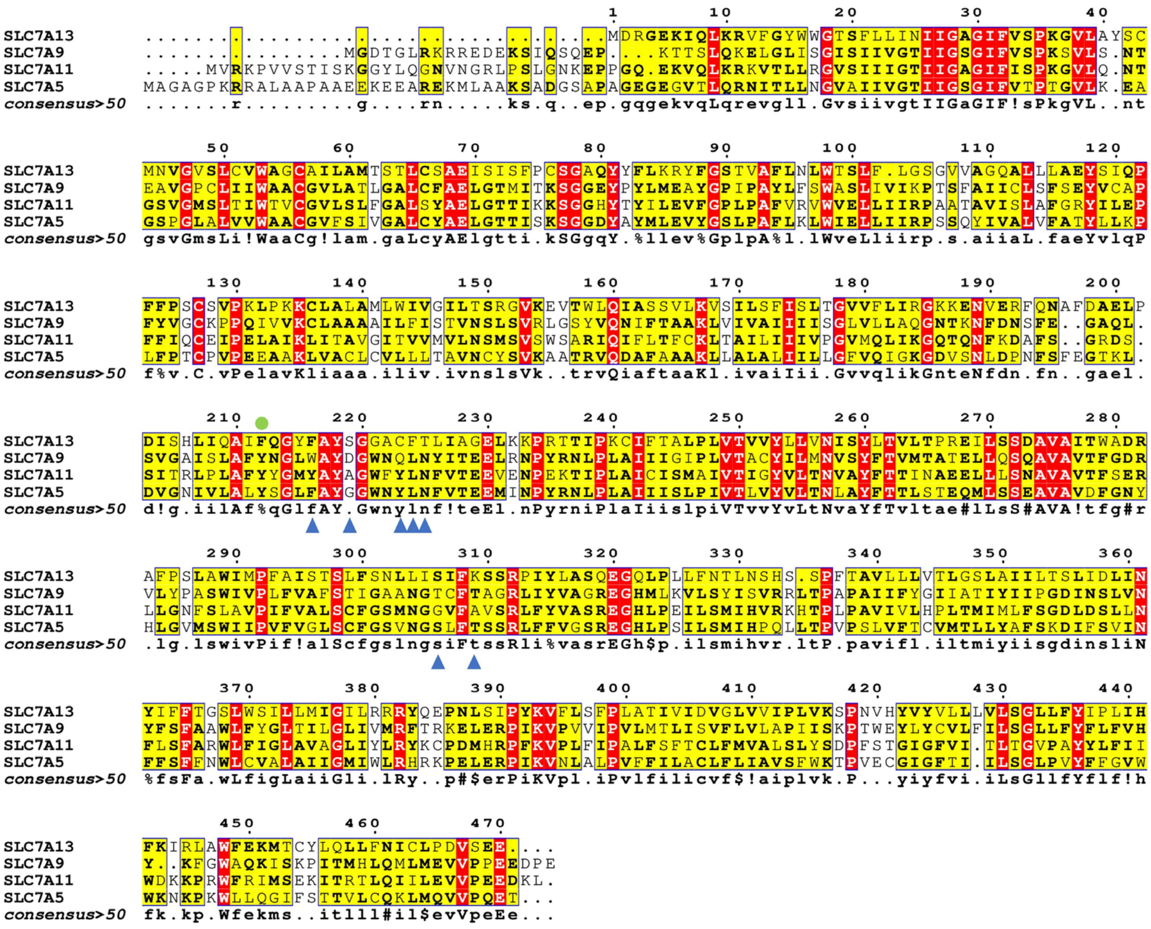

Fig. S7. Sequence alignment of SLC7A13 homologs. The sequences of SLC7A13, SLC7A9, SLC7A11, and SLC7A5 from *Homo sapiens* are aligned using the website of Mult Alin. Amino acids that are invariant and highly conserved are colored red and yellow, respectively. The blue triangles represent amino acid mutation sites constructed in SLC7A13, and the green circles represent gating residues.

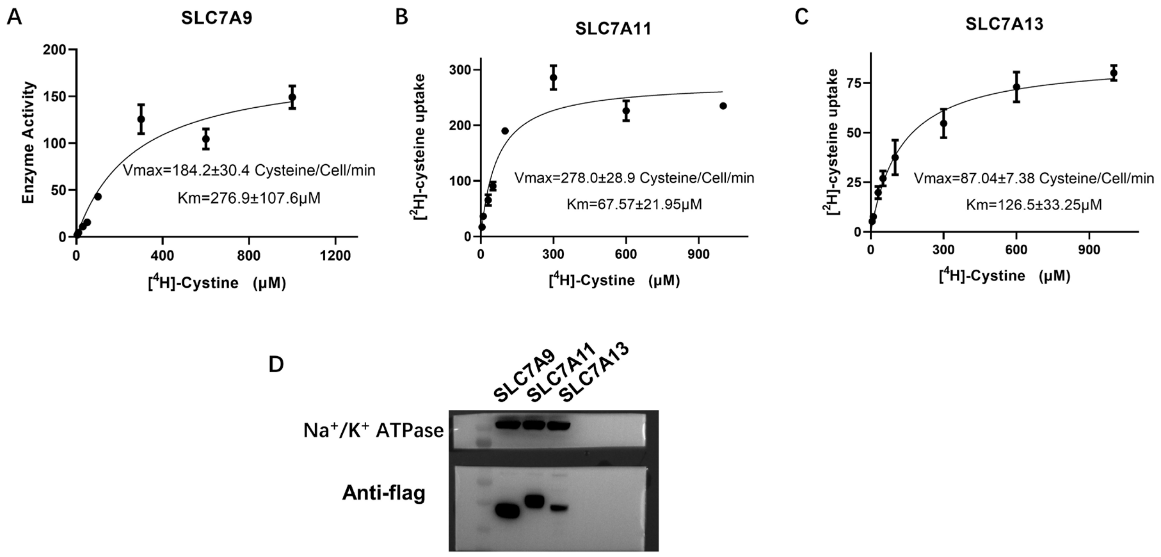

Fig. S8. Cell-based transport kinetic properties of SLC7A9 (A), SLC7A13 (B), and SLC7A11 (C)-mediated concentration-dependent cystine uptake. Data points were fitted with a Michaelis-Menten equation. Each data point had three replicates. Data are presented as mean ± SD. (D) Detection of SLC7A9, SLC7A11, and SLC7A13 protein expression using western blotting in transfected cells, with Na+/K+ ATPase as a loading control.

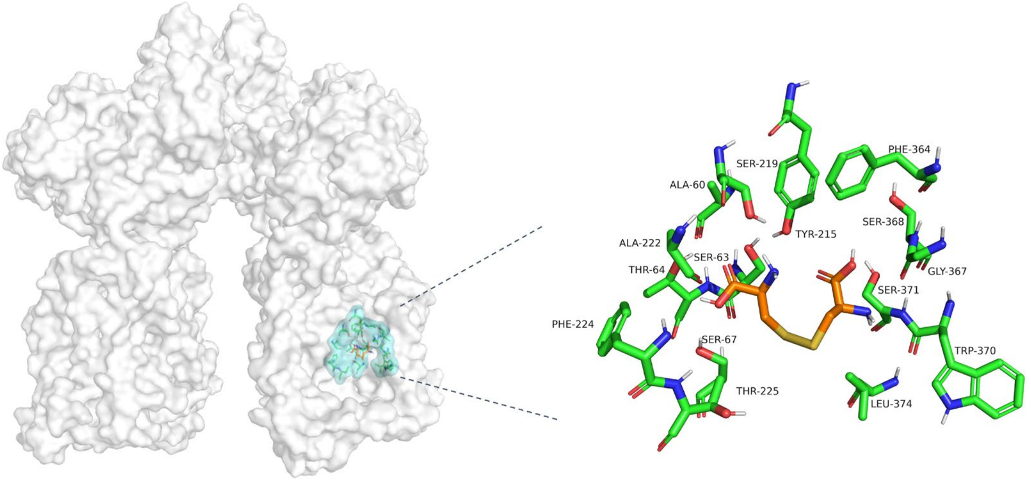

Fig. S9. Molecular docking of cystine binding mode in SLC7A13. The pocket residues are shown as sticks.

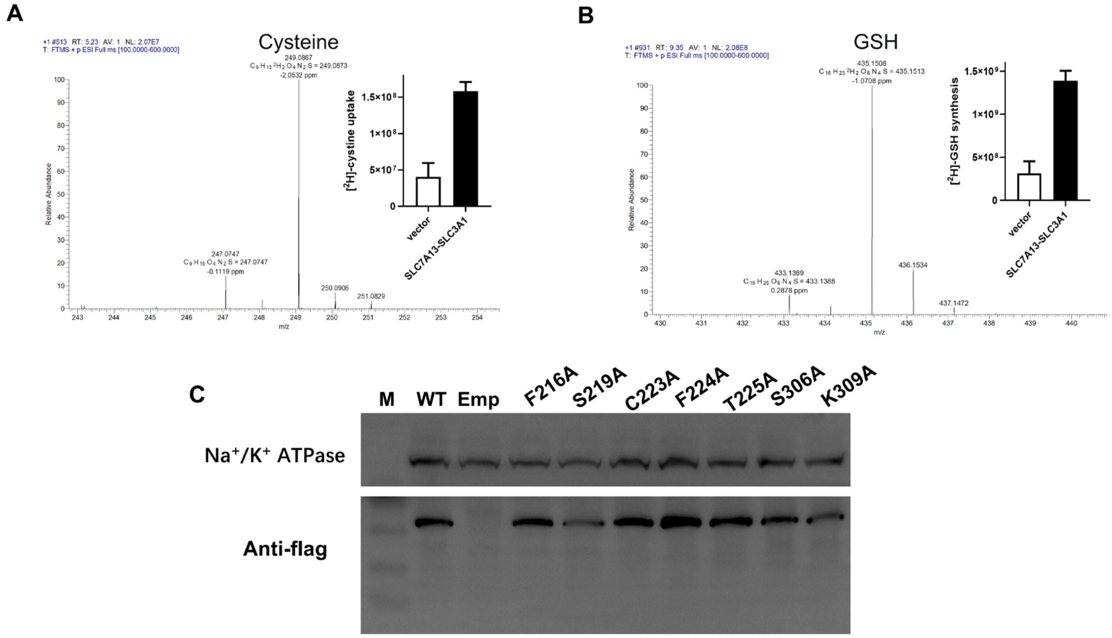

Fig. S10. Cell-based transport assays of stable isotope mass spectrometry. (A, B) The original time-of-flight secondary ion mass spectrometry (ToF-SIMS) mass spectrum (positive mode) of [^{2}H] cysteine and [^{2}H] GSH. Inset, overexpression of the SLC7A13- SLC3A1 complex augments cystine uptake and GSH content. **(C)** WB detection of SLC7A13 mutations and wild type were expressed in cells to a comparable level. Na+/K+ ATPase was used as the loading control.

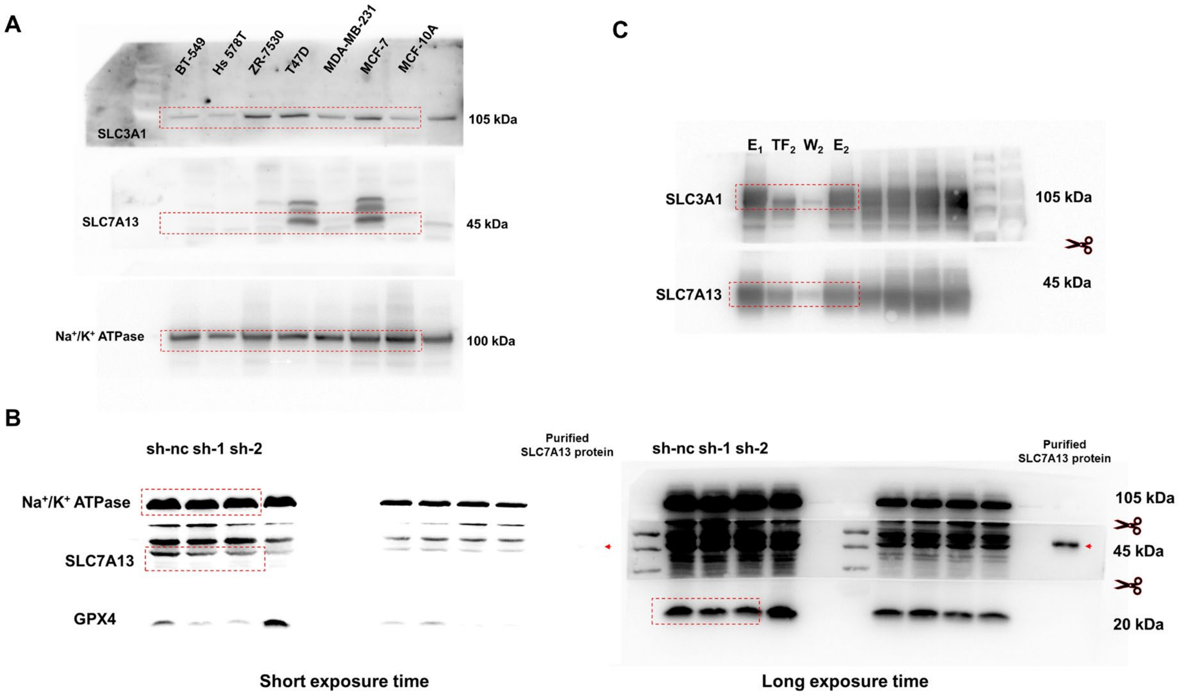

Fig. S11. Images of full western blots for each WB image used in figures, including those of Figure 2A **(A),** Figure 3D **(B)** (images at left and right were taken with different exposure times), and Figure S2B **(C)**. The area framed by dotted lines indicates the WB image used in the figures.

Table S1. Data collection, 3D reconstruction, and model statistics

**Data collection**	
EM equipment	Titan Krios (Thermo Fisher Scientific)
Voltage(kV)	300
Detector	Gatan K3 Summit
Energy filter	Gatan GIF Quantum, 20 eV slit
Pixel size(Å)	1.095
Electron dose(e-/Å2)	50
Defocus range(μm)	-1.3 ~ -1.8
Number of collected micrographs	6619
**3D Reconstruction**	
Software	cryoSPARC
Number of used particles (Overall)	462,604
Resolution(Å)	2.64
Symmetry	C2
Map sharpening B-factor (Å2)	-119.2
**Refinement**	
Software	Phenix
Cell dimensions	
a=b=c(Å)	280.32
α=β=γ(°)	90
Model composition	
Protein residues	2144
Side chains assigned	2144
Sugar	20
Ca^{2+}	2
R.m.s deviations	
Bonds length(Å)	0.002
Bond Angle(°)	0.511
Ramachandran plot statistics(%)	
Preferred	97.8
Allowed	2.2
Otlier	0

References

CHANRION, M., NEGRE, V., FONTAINE, H., SALVETAT, N., BIBEAU, F., MAC GROGAN, G., MAURIAC, L., KATSAROS, D., MOLINA, F., THEILLET, C. & DARBON, J. M. 2008. A gene expression signature that can predict the recurrence of tamoxifen-treated primary breast cancer. *Clin Cancer Res,* 14**,** 1744-52.

CURTIS, C., SHAH, S. P., CHIN, S. F., TURASHVILI, G., RUEDA, O. M., DUNNING, M. J., SPEED, D., LYNCH, A. G., SAMARAJIWA, S., YUAN, Y., GRäF, S., HA, G., HAFFARI, G., BASHASHATI, A., RUSSELL, R., MCKINNEY, S., LANGERøD, A., GREEN, A., PROVENZANO, E., WISHART, G., PINDER, S., WATSON, P., MARKOWETZ, F., MURPHY, L., ELLIS, I., PURUSHOTHAM, A., BøRRESEN-DALE, A. L., BRENTON, J. D., TAVARé, S., CALDAS, C. & APARICIO, S. 2012. The genomic and transcriptomic architecture of 2,000 breast tumours reveals novel subgroups. *Nature,* 486**,** 346-52.

PEREIRA, B., CHIN, S. F., RUEDA, O. M., VOLLAN, H. K., PROVENZANO, E., BARDWELL, H. A., PUGH, M., JONES, L., RUSSELL, R., SAMMUT, S. J., TSUI, D. W., LIU, B., DAWSON, S. J., ABRAHAM, J., NORTHEN, H., PEDEN, J. F., MUKHERJEE, A., TURASHVILI, G., GREEN, A. R., MCKINNEY, S., OLOUMI, A., SHAH, S., ROSENFELD, N., MURPHY, L., BENTLEY, D. R., ELLIS, I. O., PURUSHOTHAM, A., PINDER, S. E., BøRRESEN-DALE, A. L., EARL, H. M., PHAROAH, P. D., ROSS, M. T., APARICIO, S. & CALDAS, C. 2016. The somatic mutation profiles of 2,433 breast cancers refines their genomic and transcriptomic landscapes. *Nat Commun,* 7**,** 11479.

RUEDA, O. M., SAMMUT, S. J., SEOANE, J. A., CHIN, S. F., CASWELL-JIN, J. L., CALLARI, M., BATRA, R., PEREIRA, B., BRUNA, A., ALI, H. R., PROVENZANO, E., LIU, B., PARISIEN, M., GILLETT, C., MCKINNEY, S., GREEN, A. R., MURPHY, L., PURUSHOTHAM, A., ELLIS, I. O., PHAROAH, P. D., RUEDA, C., APARICIO, S., CALDAS, C. & CURTIS, C. 2019. Dynamics of breast-cancer relapse reveal late-recurring ER-positive genomic subgroups. *Nature,* 567**,** 399-404.

SUN, X., LI, J., DONG, F. N. & DONG, J. T. 2014. Characterization of nuclear localization and SUMOylation of the ATBF1 transcription factor in epithelial cells. *PLoS One,* 9**,** e92746.

YAN, R., LI, Y., SHI, Y., ZHOU, J., LEI, J., HUANG, J. & ZHOU, Q. 2020. Cryo-EM structure of the human heteromeric amino acid transporter b(0,+)AT-rBAT. *Sci Adv,* 6**,** eaay6379.
